# Economic burden of disease progression among multiple myeloma patients who have received transplant and at least one line of therapy in the US

**DOI:** 10.1038/s41408-021-00431-5

**Published:** 2021-02-16

**Authors:** Rafael Fonseca, May Hagiwara, Sumeet Panjabi, Emre Yucel, Jacqueline Buchanan, Thomas Delea

**Affiliations:** 1 Mayo Clinic Comprehensive Cancer Center, Phoenix, AZ USA; 2 Policy Analysis Inc., Chestnut Hill, MA USA; 3 Amgen Inc., South San Francisco, CA USA; 4 Amgen Inc., Thousand Oaks, CA USA

**Keywords:** Health services, Cancer

## Abstract

Effects of disease progression on healthcare resource utilization (HRU) and costs among multiple myeloma (MM) patients with ≥1 line of therapy (LOT) who received their first stem cell transplant (SCT) within 1 year of initial MM diagnosis were estimated using a large US claims database. Disease progression was defined as advancement to the next LOT, bone metastasis, hypercalcemia, soft tissue plasmacytoma, skeletal related events, acute kidney disease, or death within 12 months of LOT initiation. Annual HRU and costs in the first three LOTs (L1–L3) were compared for patients with versus without disease progression using inverse probability of treatment weighting to adjust for differences between groups in baseline characteristics. In all LOTs, mean annual hospitalizations and healthcare costs were greater for patients with versus without progression. Total incremental annual costs among patients with versus without progression in L1–L3 were $18,359, $87,055, and $71,917, respectively, among LOTs initiated between 2006 and 2018. In LOTs initiated between 2013 and 2018, the figures were $46,024, $100,329, and $101,942 in L1–L3, respectively. The economic burden of disease progression is substantial in this population of MM patients who underwent SCT and received systemic anti-myeloma therapy.

## Introduction

Multiple Myeloma (MM) is a rare hematologic malignancy of the plasma cells that is associated with a variety of complications, including—but not limited to—anemia, neutropenia, thrombocytopenia, bone loss and fractures, and kidney disease. In the US, ~32,270 new cases of MM are diagnosed, and ~12,830 persons die from the disease each year. Five-year survival for patients diagnosed with MM is ~54%^1^.

Current treatment guidelines by the National Comprehensive Cancer Network (NCCN) and the International Myeloma Working Group recommend initiating therapy when patients become symptomatic or are identified based on biomarkers of malignancy^2,3^. Initial treatment for transplant eligible (TE) patients is individualized according to patients’ risk factors^2^. Most patients receive an initial regimen that includes combination treatment with a corticosteroid (i.e., dexamethasone) and a proteasome inhibitor (PI: most commonly, bortezomib) and an immunomodulatory drug (IMiD: most commonly lenalidomide), and there is an emerging role for monoclonal antibodies in quadruplet regimens (mAb: primarily daratumumab), and/or a chemotherapy^2^. For patients who are refractory to or relapse following initial treatment, the NCCN Guidelines recommend numerous treatment options including PIs, IMiDs, and mAbs^2^. Several other drugs have been recently approved for their use in relapsed and refractory MM including selinexor^4^ and belantamab^5^. Despite the multitude of options, the likelihood and duration of response decrease with each line of therapy (LOT) and the disease is ultimately fatal for many patients^2^.

Previous retrospective observational studies have documented substantial costs for MM patients receiving second (i.e., second line) or subsequent treatments^6–13^, focusing on patients who are progressing and need to receive further lines of therapy. However, evidence on economic burden specifically focusing on MM patients who received transplant is lacking. Hagiwara^13^ reported, among transplant ineligible MM patients who initiated LOTs between 2006 and 2016, total annual healthcare costs were greater for patients with versus without progression, with a difference of $25,920, $30,632, $47,320, and $19,769 for LOTs 1–4 (L1–L4), respectively. The objective of this study was to investigate if the results found among *transplant ineligible* MM patients in Hagiwara^13^ can also be found among MM patients with receipt of transplant using the same approach as much as possible except for the algorithm for identifying LOTs: i.e., to compare and quantify HRU and healthcare costs in the US between MM patients with versus without progression who have received at least one LOT including a stem cell transplant using inverse probability of treatment weights (IPTW) to adjust for differences between the two groups^14^.

## Methods

### Study design and patient selection

A retrospective incident user cohort design was employed^15^. Data for the study were obtained from the IBM MarketScan^®^ Commercial Claims and Encounters (CCAE) and Medicare and Coordination of Benefits (MDCR) databases. Study subjects included all adult patients in the databases with a confirmed diagnosis of MM during the study period (January 1, 2006 through September 30, 2018), where a confirmed diagnosis of MM was defined as at least one inpatient claim with a diagnosis of MM (ICD-9-CM code 203.0x or ICD-10-CM code C90.0), or at least two nondiagnostic outpatient claims within 30 to 365 days apart with a diagnosis of MM. The date of each patient’s first claim with a diagnosis of MM was designated the “diagnosis date”. To ensure that the first claim with a diagnosis of MM in the databases represented initial diagnosis of the disease, patients with <6 months of continuous enrollment prior to their diagnosis date were excluded. Patients were further required to have initiated during the study period a course of MM therapy including one or more of the following: lenalidomide, pomalidomide, thalidomide, bortezomib, carfilzomib, ixazomib, daratumumab, elotuzumab, panobinostat, or vorinostat, and have received stem cell transplant (SCT) within 1 year of the diagnosis date. Although vorinostat is not indicated for MM, it was included in our study since its use among MM patients has been investigated in clinical studies^16^. The following patients were excluded: patients with receipt of MM therapy or unclassified antineoplastic medications (HCPCS Code J9999) before the diagnosis date; patients with evidence of receipt of more than one type of PIs simultaneously; patients with missing information on supply amounts of MM therapy that could not be imputed; and patients with missing demographic information.

### Data source

The CCAE and MDCR databases contain enrollment information and information on the health insurance claims of employees of large, self-insured corporations and their dependents, along with a few commercial health plans (CCAE) and for Medicare-eligible persons (mainly retirees) who are also covered by self-insured employers (MDCR), and they are fully de-identified and compliant with the Health Insurance Portability and Accountability Act of 1996^17^. All claims include information on costs for adjudicated claims including amounts paid by insurers and patients. For each person in the databases, claim-level data can be arrayed in chronologic order to provide a detailed, longitudinal profile of all medical and pharmacy services received. The databases have been used widely in health services research over the past decade.

### Identification of LOTs

The algorithm for identifying LOTs was determined based on a previously published algorithm for transplant ineligible patients^18^ that was adapted for TE patients based on direct review of the temporal pattern of claims for MM treatments by a clinical expert on the study team (Dr. Fonseca, Mayo Clinic, Phoenix Arizona, US). For each qualified patient, the date of initiation of L1 was defined based on the date of first receipt of MM therapies before the date of the first SCT claim. Patients who had evidence of a second SCT within 1 year of the initial SCT were assumed to have received tandem SCT. For each patient, the date of L2 initiation was determined by the date of the first MM therapy received after SCTs as described in the Appendix. For L2 onward, the date of the next LOT was based on the earliest of: (1) the date of the first claim for a “*new* MM therapy,” defined as a MM therapy for which there was no claim during the treatment identification period; (2) the date of the first claim for any MM therapy with a gap period greater than 90 days after the end of cycle; or (3) for patients who received more than one type of PI during the treatment identification period, the first date with a claim for the second type of PI. Only the first three LOTs were considered in the analysis as the number of patients with more than three LOTs was relatively small.

### Disease progression and matching patients

**Fig. 1:**
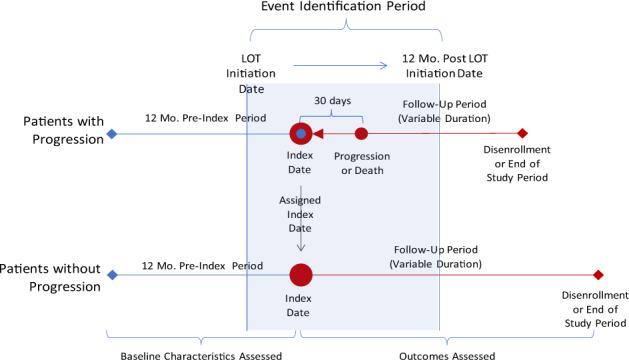
Study design. For each LOT, progression status was assessed based on claims observed during an “event identification period”, defined as the first 12-month period after LOT initiation date (small red circle). For patients with progression, the “index date” (large red circle) was defined as the date 30 days prior to documented progression (in order to capture care during the “prodromal” period and terminal care for those who died). For patients without progression, index date was randomly assigned to ensure similar distribution of time from treatment initiation to index date as for patients with progression. Footnote: Event = Progression or death. LOT = Line of therapy. Mo = Month.

For each qualified patient and LOT, progression status was assessed based on claims observed during an “event identification period”, defined as the first 12-month period after LOT initiation date (Fig. 1). Disease progression was defined as: (1) advancement to the next LOT; (2) evidence of bone metastasis, hypercalcemia, soft tissue plasmacytoma, skeletal related events, fractures, acute kidney disease, and renal failure while off-therapy; or (3) evidence of death (based on discharge status, diagnosis codes, and receipt of hospice care that are observed within 1 month of disenrollment, excluding the disenrollment date of September 30, 2018, the end of study period). For patients with progression, the “index date” was defined as the date 30 days prior to documented progression (in order to capture care during “prodromal” period and terminal care for those who died). For patients without progression, index date was randomly assigned to ensure similar distribution of time from treatment initiation to index date as for patients with progression. Patient baseline clinical characteristics were assessed during “pre-index period”, defined as 12 months prior to index date. Patients with less than 12 months of continuous enrollment pre-index were excluded. Outcomes of interest were examined over the “follow-up period” defined as the period beginning with the index date and ending with the end of the study period (September 30, 2018) or the end of post-index continuous enrollment, whichever occurred first. For patients with claims for unclassified antineoplastic medications, the follow-up period was truncated on the date of the claim. When patients on Commercial plan switched to Medicare plan, the follow-up period was truncated at the date of the switch. Analyzes were conducted by LOT; thus, each patient could contribute to multiple analyses.

### Outcomes and analysis

Annual HRU and total healthcare costs during the follow-up period were calculated for each patient and LOT. HRU measures included number of emergency department visits, physicians’ office and outpatient hospital visits, other outpatient visits, outpatient prescription claims, inpatient admissions, and inpatient days. Cost measures included costs of MM therapy (including medication costs and costs of administration), costs of SCT, costs of inpatient hospitalization not associated with SCT, costs of outpatient services excluding services for SCT or MM therapy, costs of outpatient pharmacy excluding MM therapy, and total costs. The cost categories were assessed hierarchically in the order described above. Costs were based on total paid amounts including amounts paid by insurers and patients (i.e., patient contributions were included). Costs were adjusted to 2018 US dollars using consumer price index for medical care published by Bureau of Labor and Statistics.

Annual HRU and costs were compared between patients with versus without progression using stabilized inverse probability of treatment weights (sIPTW)^14^ to adjust for differences between the two groups in baseline characteristics including year of index date, age, gender, region, plan type, Medicare status, comorbidities, and pre-index HRU and healthcare costs. For each LOT, IPTWs were estimated using logistic regression with the progression status as the dependent variable and baseline patient characteristics as independent variables. The resulting predicted probability of progression was used to calculate the weights. Average treatment effect (ATE) weights were used. Stabilized weights were calculated using the marginal probabilities of progression and no progression. To adjust for difference in duration of follow-up period, individual patients were also weighted by duration of follow-up. *P* values were calculated using *t*-test with stabilized IPTW, and the 95% confidence intervals (CIs) were calculated based on the bootstrap method with 2000 replicated samples.

For each LOT, expected cumulative total *plan* costs for patients with versus without progression were calculated using the Kaplan–Meier sampling average (KMSA) method^19^, with disenrollment used in place of death. In these analyzes, patients were censored when their disenrollment occurred at the end of the study period. To evaluate temporal trends, a subgroup analysis was conducted for LOTs initiated before and after 2013.

## Results

### Study patients

**Fig. 2:**
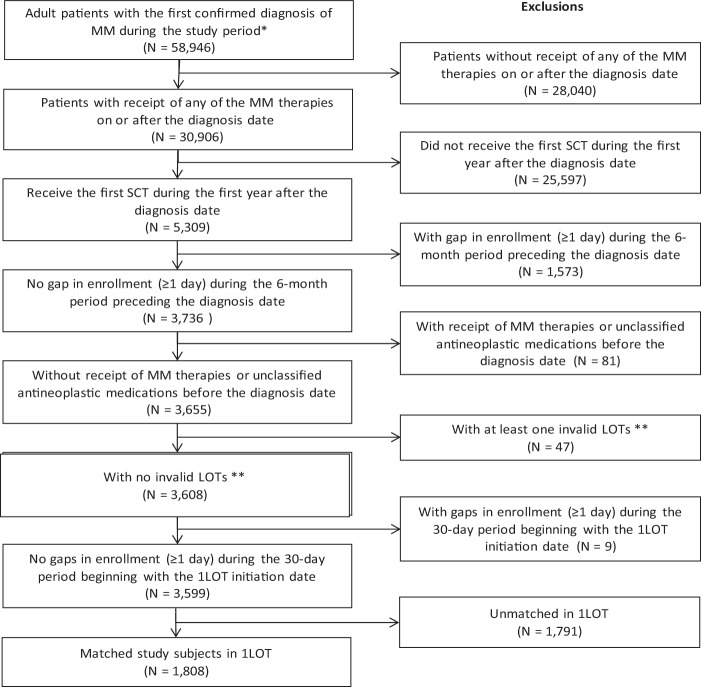
Patient selection. Footnote: LOT = Line of therapy. MM = Multiple myeloma. SCT = Stem cell transplant. *Confirmed diagnosis is defined as at least one inpatient claim, or at least two non-diagnostic outpatient claims within 30 to 365 days apart with a diagnosis of MM (ICD-9-CM code 203.0x or ICD-10-CM code C90.0). **Missing information on supply amounts of MM therapy that could not be imputed, or at least one day with evidence of receipt of multiple proteasome inhibitors simultaneously.

Of the 5309 adult MM patients who received one or more MM therapies (including a stem cell transplant) during the study period, 3599 qualified for the study, including 934 patients with progression and 2665 without progression during L1, respectively (Fig. 2). The numbers of matched pairs of patients with and without progression were 904 in L1, 482 in L2, and 244 in L3.

### Baseline characteristics

**Fig. 3:**
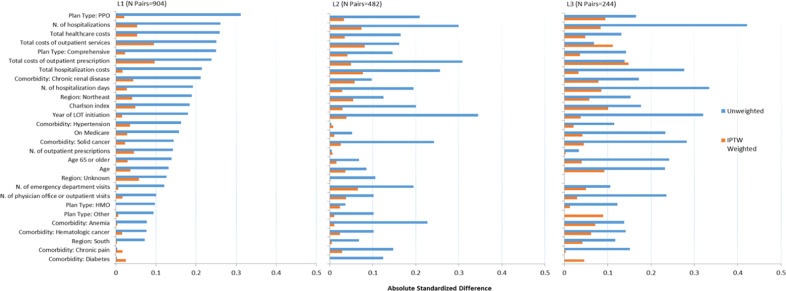
Standardized Differences in Patient Characteristics. Differences among patients with versus without progression based on unweighted and sIPTW analyses by LOT (unweighted standardized difference 0.1 or more in any LOT).

Mean age at index date was 58 years, and 56% were male (Table 1). In all LOTs, patients with progression had more comorbidities than those without progression. Number of emergency department visits, physicians’ office and other outpatient visits, and hospitalizations during the 12-month period before index date (date of progression) were all higher among patients with progression in all LOTs. Hospitalization costs and costs of outpatient services during the pre-index period were higher among patients with progression, but the cost of outpatient prescription during the same period was higher among patients without progression. With sIPTW adjustment, all baseline characteristics were well balanced with standardized mean differences (SMD) < 0.1 for all characteristics for L1 and L2. For L3, SMDs were <0.1 for most of the baseline characteristics except for baseline costs for outpatient services and prescriptions, and Charlson index (Fig. 3).

**Table 1:** Patient characteristics at index date by progression and LOT (for matched patients).

Characteristic	L1		L2		L3	
	Without progression	With progression	Without progression	With progression	Without progression	With progression
	(*N* = 904)	(*N* = 904)	(*N* = 482)	(*N* = 482)	(*N* = 244)	(*N* = 244)
Age, year, mean(SD)	58.8 (8.1)	57.7 (8.1)	59.7 (8.4)	59.0 (8.3)	61.0 (8.7)	59.0 (8.3)
Females, N. (%)	410 (45.4)	370 (40.9)	217 (45.0)	207 (42.9)	108 (44.3)	96 (39.3)
On Medicare, N. (%)	194 (21.5)	139 (15.4)	102 (21.2)	92 (19.1)	63 (25.8)	40 (16.4)
Charlson index, mean (SD)^a^	1.25 (1.46)	1.53 (1.60)	1.01 (1.29)	1.30 (1.55)	1.25 (1.45)	1.54 (1.79)
Healthcare utilization, mean (SD)^a^						
N. of ED visits	1.35 (2.07)	1.66 (3.06)	0.87 (1.55)	1.51 (4.42)	1.19 (3.46)	1.53 (2.86)
N. of PO or OP visits	35.5 (20.9)	37.6 (20.9)	30.9 (20.9)	33.2 (23.8)	31.4 (21.7)	37.1 (27.0)
N. of OP Rx	50.3 (25.6)	46.0 (34.5)	51.4 (27.9)	51.5 (35.8)	53.5 (31.0)	52.4 (33.8)
N. of hospitalizations	1.44 (1.32)	1.84 (1.69)	0.79 (1.19)	1.22 (1.62)	0.79 (1.29)	1.43 (1.72)
N. of inpatient days	13.5 (18.2)	17.1 (19.4)	7.5 (13.9)	10.5 (16.8)	6.0 (11.9)	10.9 (17.3)
Healthcare costs, $ in 2018 mean (SD)^a^						
OP services	111,467 (90,502)	136,146 (105,538)	100,332 (127,078)	119,756 (113,076)	126,469 (147,263)	135,429 (115,274)
OP prescriptions	33,572 (24,975)	27,279 (27,648)	75,422 (53,026)	59,226 (52,013)	79,956 (63,438)	71,575 (57,123)
Inpatient Rx	79,295 (102,333)	104,748 (133,505)	43,009 (79,829)	68,174 (113,674)	36,577 (73,974)	60,034 (94,580)
Total healthcare costs	224,334 (149,795)	268,172 (187,016)	218,763 (167,353)	247,156 (176,767)	243,002 (189,773)	267,038 (175,016)

^a^Calculated over 12-month period prior to the index date.

*ED* emergency department, *LOT* line of therapy, *N* number, *OP* outpatient, *PO* physician office. *Rx* prescription, *SD* standard deviation

### Healthcare utilization and costs

**Fig. 4:**
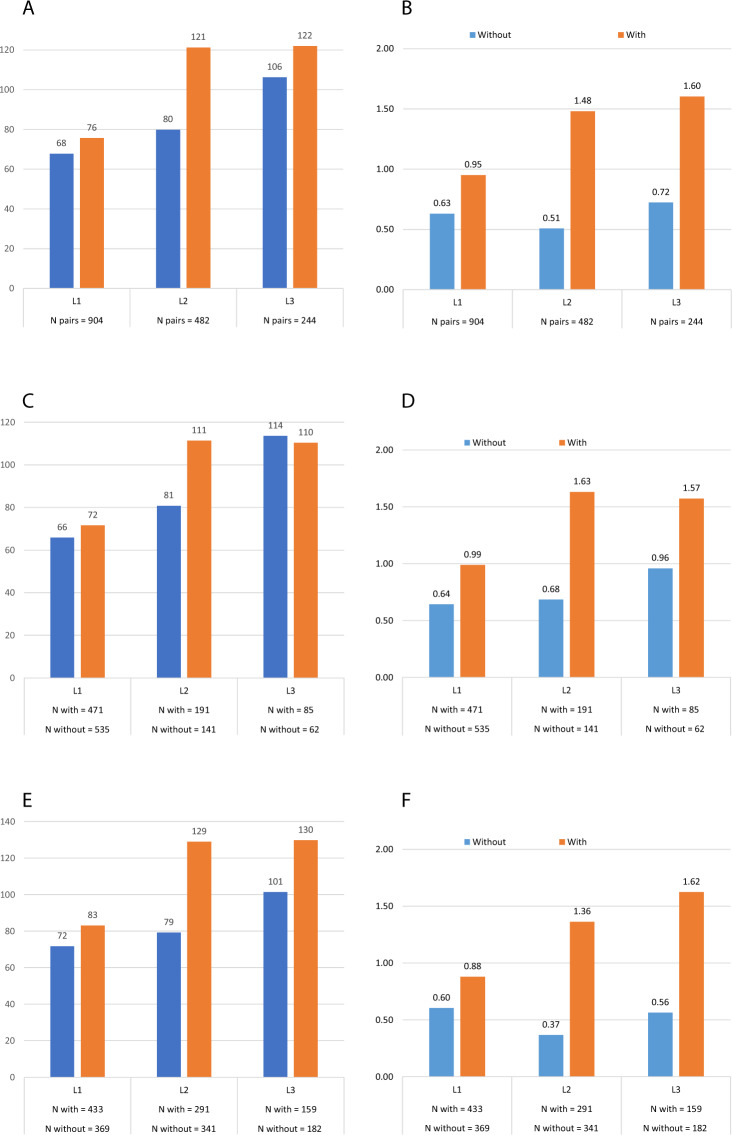
IPTW adjusted annual healthcare resource utilization among patients with versus without progression by LOT. **A** Outpatient visits between 2006 and 2018; **B** Hospitalization between 2006 and 2018; **C** Outpatient visits between 2006 and 2012; **D** Hospitalization between 2006 and 2012; **E** Outpatient visits between 2013 and 2018; **F** Hospitalization between 2013 and 2018.

**Fig. 5:**
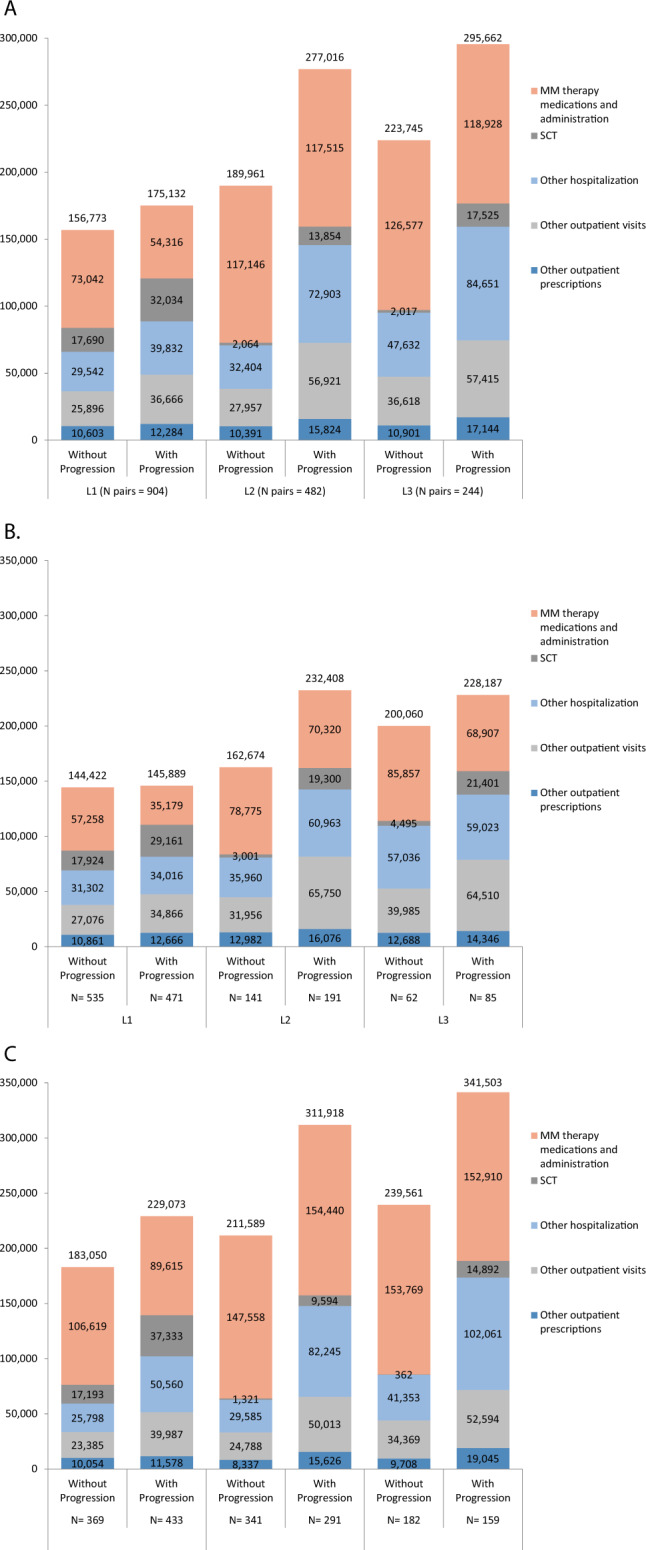
IPTW adjusted mean annual healthcare costs among patients with versus without progression by LOT. **A** LOTs initiated between 2006 to 2018; **B** LOTs initiated between 2006 to 2012; **C** LOTs initiated between 2013 to 2018.

The mean number of hospitalizations per year was greater for patients with versus without progression in all LOTs, with differences ranging from 0.97 (95%CI 0.73, 1.22) for L2 to 0.32 (95%CI 0.20, 0.44) for L1 (Table 2 and Fig. 4A, B). The mean number of outpatient visits per year also was greater for patients with versus without progression in L1 through L3. With the exception of L2, annual MM therapy costs were lower for patients with versus without progression (Table 2 and Fig. 5A). Annual costs of outpatient services, excluding visits associated with MM therapy and hospitalization, were greater for patients with versus without progression for all LOTs. Total annual healthcare costs were greater for patients with versus without progression in all LOTs with the incremental annual costs (95%CI) of $18,359 ($2289–$34,429), $87,055 ($61,778–$112,333), and $71,917 ($36,452–$107,382) in L1, L2, and L3, respectively. These incremental annual costs for patients with versus without progression were mostly MM-related: $16,678 (91%), $81,622 (94%), and $65,674 (91%) in L1, L2, and L3, respectively.

**Table 2:** Mean (SE) annual healthcare resource utilization and costs during the follow-up period, for IPTW matched sample of ptspatients with vsversus without progression by LOT and index year among TE MM patients.

LOT	Outcome	LOT initiated in year 2006–2018			LOT initiated in year 2006–2012			LOT initiated in year 2013–2018		
		Without progression	With progression	Difference (95% CI)	Without progression	With progression	Difference (95% CI)	Without progression	With progression	Difference (95% CI)
1	*N* unweighted (weighted)	904 (847)	904 (907)		535 (470)	471 (502)		369 (377)	433 (405)	
	Follow-up months, Mean (SE)	31.98 (0.89)	22.02 (0.76)		39.22 (1.33)	25.80 (1.23)		22.96 (0.86)	17.33 (0.77)	
	Outpatient visits, Mean (SE) per y	67.75 (1.41)	75.65 (1.74)	7.90 (3.51, 12.30)***	65.85 (1.87)	71.60 (2.33)	5.74 (−0.13, 11.62)	71.78 (2.11)	83.13 (2.61)	11.35 (4.75, 17.94)***
	Hospitalizations, Mean (SE) per y	0.630 (0.03)	0.950 (0.05)	0.32 (0.20, 0.44)***	0.642 (0.04)	0.989 (0.07)	0.35 (0.19, 0.51)***	0.604 (0.05)	0.879 (0.08)	0.28 (0.10, 0.46)**
	Length of stay at hospital, Mean (SE) per y	5.569 (0.74)	10.128 (0.72)	4.56 (2.54, 6.58)***	5.663 (1.10)	9.943 (0.93)	4.28 (1.45, 7.11)**	5.369 (0.65)	10.470 (1.17)	5.10 (2.48, 7.72)***
	Costs, Mean (SE) $ per y									
	MM therapy medications and administration	73,042 (2111)	54,316 (2552)	−18,726 (−25,221, −12,231)***	57,258 (2028)	35,179 (2331)	−22,080 (−28,144, −16,016)***	106,619 (4120)	89,615 (4834)	−17,004 (−29,472, −4536)**
	Stem cell transplant	17,690 (3360)	32,034 (3297)	14,344 (5112, 23,576)**	17,924 (5,121)	29,161 (3512)	11,237 (−950, 23,424)	17,193 (2380)	37,333 (6306)	20,140 (6901, 33,379)**
	Other hospitalization	15,018 (1178)	25,129 (2774)	10,111 (4199, 16,023)***	15,282 (1326)	24,883 (4220)	9601 (912, 18,289)*	14,457 (2287)	25,583 (3162)	11,126 (3466, 18,787)**
	Other outpatient visits	45,633 (1946)	57,514 (2885)	11,880 (5054, 18,707)***	48,272 (2780)	50,286 (2935)	2015 (−5919, 9949)	40,020 (2252)	70,845 (5619)	30,824 (18,934, 42,715)***
	Other outpatient prescriptions	5389 (304)	6139 (358)	750 (−171, 1671)	5685 (388)	6379 (465)	694 (−495, 1883)	4760 (494)	5697 (570)	937 (−544, 2418)
	Total	156,773 (5004)	175,132 (6488)	18,359 (2289, 34,429)*	144,422 (6811)	145,889 (7701)	1,467 (−18,708, 21,642)	183,050 (6798)	229,073 (10,993)	46,024 (20,647, 71,400)***
2	*N* unweighted (weighted)	482 (469)	482 (478)		141 (151)	191 (164)		341 (318)	291 (315)	
	Follow-up months, Mean (SE)	18.75 (0.95)	14.31 (0.77)		25.80 (2.39)	18.37 (1.74)		15.42 (0.81)	12.20 (0.65)	
	Outpatient visits, Mean (SE) per y	79.92 (2.55)	121.22 (3.28)	41.30 (33.14, 49.46)***	80.84 (4.52)	111.32 (4.90)	30.49 (17.36, 43.61)***	79.20 (3.14)	128.97 (4.36)	49.77 (39.22, 60.32)***
	Hospitalizations, Mean (SE) per y	0.507 (0.06)	1.481 (0.11)	0.97 (0.73, 1.22)***	0.685 (0.15)	1.632 (0.18)	0.95 (0.50, 1.40)***	0.366 (0.04)	1.363 (0.13)	1.00 (0.72, 1.28)***
	Length of stay at hospital, Mean (SE) per y	2.951 (0.38)	11.248 (1.00)	8.30 (6.20, 10.40)***	4.043 (0.80)	13.034 (1.78)	8.99 (5.15, 12.84)***	2.086 (0.40)	9.850 (1.14)	7.76 (5.39, 10.14)***
	Costs, Mean (SE) $ per y									
	MM therapy medications and administration	117,146 (4291)	117,515 (4837)	369 (−12,321, 13,059)	78,775 (5325)	70,320 (3818)	−8455 (−21,355, 4446)	147,558 (5594)	154,440 (7140)	6881 (−10,935, 24,698)
	Stem cell transplant	2064 (497)	13,854 (4084)	11,790 (3,706, 19,874)**	3001 (986)	19,300 (8263)	16,298 (−114, 32,710)	1321 (552)	9594 (3765)	8273 (784, 15,762)*
	Other hospitalization	14,152 (1761)	50,664 (5382)	36,512 (25,389, 47,634)***	17,332 (3841)	51,006 (9427)	33,674 (13,625, 53,723)**	11,632 (1733)	50,396 (6332)	38,764 (25,850, 51,678)***
	Other outpatient visits	51,167 (4673)	86,680 (5002)	35,513 (22,080, 48,945)***	56,172 (7818)	84,526 (7217)	28,354 (7419, 49,288)**	47,200 (5943)	88,365 (6873)	41,165 (23,320, 59,009)***
	Other outpatient prescriptions	5432 (330)	8304 (779)	2872 (1211, 4532)***	7394 (745)	7257 (687)	−137 (−2131, 1856)	3877 (282)	9123 (1243)	5246 (2739, 7753)***
	Total	189,961 (7028)	277,016 (10,791)	87,055 (61,778, 112,333)***	162,674 (10,917)	232,408 (16,855)	69,734 (30,221, 109,247)***	211,589 (9125)	311,918 (13,750)	100,329 (67,909, 132,748)***
3	*N* unweighted (weighted)	244 (248)	244 (243)		62 (77)	85 (71)		182 (171)	159 (172)	
	Follow-up months, Mean (SE)	11.80 (0.99)	12.84 (1.02)		15.25 (2.73)	17.79 (2.51)		10.25 (0.85)	10.80 (0.88)	
	Outpatient visits, Mean (SE) per y	106.24 (4.93)	121.95 (4.63)	15.70 (2.40, 29.01)*	113.56 (10.67)	110.35 (7.08)	−3.21 (−28.59, 22.17)	101.36 (5.34)	129.83 (6.03)	28.47 (12.62, 44.32)***
	Hospitalizations, Mean (SE) per y	0.722 (0.10)	1.604 (0.17)	0.88 (0.50, 1.26)***	0.959 (0.17)	1.574 (0.27)	0.61 (−0.02, 1.25)	0.565 (0.12)	1.624 (0.21)	1.06 (0.58, 1.54)***
	Length of stay at hospital, Mean (SE) per y	3.983 (0.69)	12.341 (1.47)	8.36 (5.16, 11.56)***	5.520 (1.49)	12.031 (2.17)	6.51 (1.31, 11.71)*	2.957 (0.75)	12.552 (1.97)	9.59 (5.44, 13.75)***
	Costs, Mean (SE) $ per y									
	MM therapy medications and administration	126,577 (6522)	118,928 (7776)	−7650 (−27,592, 12,292)	85,857 (7706)	68,907 (6213)	−16,950 (−36,537, 2637)	153,769 (8466)	152,910 (11,142)	−859 (−28,395, 26,678)
	Stem cell transplant	2017 (1193)	17,525 (4826)	15,509 (5722, 25,295)**	4495 (3721)	21,401 (7445)	16,906 (428, 33,384)*	362 (175)	14,892 (6326)	14,530 (2030, 27,030)*
	Other hospitalization	19,976 (4128)	55,591 (7799)	35,616 (18,263, 52,968)***	21,254 (6244)	45,607 (10,791)	24,353 (−312, 49,019)	19,122 (5420)	62,374 (10,692)	43,252 (19,636, 66,868)***
	Other outpatient visits	67,404 (5586)	92,868 (7315)	25,464 (7,376, 43,551)**	78,810 (10,821)	84,377 (11,239)	5567 (−25,274, 36,407)	59,787 (6536)	98,636 (9594)	38,849 (15,999, 61,699)***
	Other outpatient prescriptions	7772 (1069)	10,751 (1594)	2979 (−793, 6751)	9645 (2390)	7895 (1129)	−1750 (−7003, 3504)	6521 (1119)	12,691 (2458)	6170 (848, 11,491)*
	Total	223,745 (10,232)	295,662 (14,863)	71,917 (36,452, 107,382)***	200,060 (19,229)	228,187 (20,968)	28,127 (−28,107, 84,360)	239,561 (12,155)	341,503 (19,464)	101,942 (56,762, 147,122)***

MM therapy includes medication and administration costs.

*p value < 0.05.

**p value < 0.01.

***p value < 0.001.

*LOS* length of stay, *LOT* line of therapy, *MM* multiple myeloma, *Rx* prescriptions, *SCT* stem cell transplant, *SE* standard error, *Y* year.

The expected cumulative total *plan* costs estimated using the KMSA method were reported every year post-index as long as at least ten patients remained in both the “with progression” and “without progression” groups. The incremental cumulative total healthcare costs among patient with versus without progression were $11,863 for L1, $68,414 for L2, and $98,507 for L3 at 1-year post-index (Table 3). At subsequent years post-index, however, patients in L1 with progression had lower costs than those without progression, while patients in L2 and L3 with progression had higher costs than those without progression.

**Table 3:** Expected cumulative total healthcare costs by progression, LOT, and years since index date.

LOT	Years since index date	LOT initiated in year 2006–2018					LOT initiated in year 2006–2012					LOT initiated in year 2013–2018				
		Without progression		With progression		Difference (95% CI)	Without progression		With progression		Difference (95% CI)	Without progression		With progression		Difference (95% CI)
		*N* at risk	Total cost	*N* at risk	Total cost		*N* at risk	Total cost	*N* at risk	Total cost		*N* at risk	Total cost	*N* at risk	Total cost	
1	1	635	180,699	489	192,562	11,863 (−4,504, 27,527)	382	185,337	294	183,918	−1,419 (−22,603, 18,325)	253	173,182	195	208,461	35,279 (5,621, 55,455)
	2	415	285,020	285	254,337	−30,683 (−53,872, −6,804)	265	289,910	173	235,673	−54,237 (−83,843, −20,773)	150	278,004	112	287,467	9,463 (−34,736, 35,313)
	3	283	356,363	168	295,424	−60,939 (−94,227, −28,625)	200	359,512	118	263,042	−96,469 (−136,364, −53,010)	83	353,290	50	354,874	1,584 (−59,038, 44,744)
	4	207	414,733	120	328,757	−85,976 (−125,630, −47,618)	164	416,447	92	288,460	−127,987 (−171,464, −82,474)	43	418,231	27	408,559	−9,671 (−93,004, 59,015)
	5	130	458,783	77	349,703	−109,080 (−153,473, −65,035)	124	464,547	69	306,244	−158,303 (−206,455, −106,117)					
	6	78	486,917	46	361,882	−125,035 (−171,931, −79,822)	78	498,301	46	317,889	−180,412 (−231,849, −126,447)					
	7	45	511,493	34	374,067	−137,427 (−187,406, −89,228)	45	527,709	34	329,598	−198,111 (−250,343, −142,544)					
	8	29	529,964	17	382,832	−147,132 (−199,665, −96,451)	29	549,810	17	338,021	−211,790 (−266,271, −154,301)					
2	1	231	162,642	189	231,055	68,414 (33,861, 98,096)	84	158,263	66	221,885	63,622 (9,389, 112,429)	147	167,537	122	235,202	67,664 (25,983, 112,440)
	2	130	262,247	80	327,744	65,496 (22,677, 107,555)	55	249,331	37	293,631	44,300 (−18,630, 110,411)	75	272,971	43	350,303	77,332 (20,738, 139,197)
	3	76	334,042	44	377,794	43,752 (−6,425, 95,870)	42	316,808	28	333,287	16,479 (−53,969, 93,575)	33	348,437	16	409,130	60,692 (−7,740, 136,101)
	4	43	378,045	19	407,728	29,684 (−29,146, 90,121)	28	357,378	17	363,422	6,044 (−73,971, 89,688)					
	5	26	415,108	11	424,741	9,633 (−56,775, 78,609)	22	392,759	11	383,496	−9,263 (−100,672, 86,829)					
3	1	81	144,123	83	242,630	98,507 (60,916, 134,759)	23	154,514	27	236,698	82,184 (4,908, 137,344)	57	132,096	56	242,348	110,252 (61,464, 156,204)
	2	42	239,126	39	333,103	93,977 (39,009, 150,407)	16	230,887	18	310,254	79,368 (−22,391, 147,610)	26	235,985	22	342,640	106,655 (33,381, 184,580)
	3	16	294,692	15	379,951	85,260 (14,737, 156,695)	11	274,648	8	349,341	74,694 (−47,010, 161,429)					

95% CI (confidence interval) was calculated based on the bootstrap method with 2000 replicated samples.

For each LOT, results are reported for years with at least ten patients at risk in both progressed and not progressed groups.

### LOTs initiated between 2006–2012 and 2013–2018

The subgroup analysis was conducted for LOTs initiated before and after 2013 to assess temporal trends in impact of progression. For LOTs initiated during the period from 2006 to 2012, the mean number of hospitalizations per year were greater for patients with versus without progression in all LOTs, and the number of outpatient visits per year were greater for patients with versus without progression in L1 and L2 (Table 2 and Fig. 4C, D). Total annual healthcare costs also were greater for patients with versus without progression for all LOTs initiated during this period, with the incremental annual costs of $1467 (95%CI $-18.708–$21,642), $69,734 (95%CI $30,221–$109,247), and $28,127 (95%CI $-28.107–$84,360) in L1 to L3, respectively (Table 2 and Fig. 5B). For LOTs initiated during the period from 2013 to 2018, the mean number of hospitalizations per year and the number of outpatient visits per year were greater for patients with versus without progression in all LOTs (Table 2 and Fig. 4E, F). Total annual healthcare costs also were greater for patients with versus without progression for all LOTs initiated during this period, with the incremental annual costs of $46,024 (95%CI $20,647–$71,400), $100,329 (95%CI $67,909–$132,748), and $101,942 (95%CI $56,762–$147,122) in L1–L3, respectively (Table 2 and Fig. 5C).

KMSA estimates of incremental cumulative total healthcare costs at 1-year post-index among patient with versus without progression were $35,279 for L1, $67,664 for L2, and $110,252 for L3 (Table 3). At subsequent years post-index, patients with progression had higher costs in all LOTs except for year 4 among L1s.

## Discussion

In this study using data from a large health insurance claims database, MM patients who received transplant and medical therapy who experienced progression within 1 year of initiating treatment had higher mean annual numbers of hospitalization and outpatient visits and higher mean annual costs and expected cumulative costs at 1 year after progression than similar patients who did not experience progression. Increased HRU and costs were observed for patients with versus without progression in all LOTs and for all outcome measures except for cost of MM therapy in L1–L3. The decrease in annual costs of MM therapy with progression in these lines may reflect unplanned therapy discontinuation as a result of encountering an event such as bone metastasis, hypercalcemia, soft tissue plasmacytoma, skeletal related events, acute kidney disease that were a part of the definition of progression.

This study is subjected to limitations inherent in the use of administrative claims data. These include misidentification of study subjects and inaccurate assessment of baseline characteristics and study outcomes due to errors in coding or lack of sensitivity and specificity of diagnosis and procedure codes for clinical outcomes. Also, it is possible that study patients may have been participating in clinical trials or patient assistance programs, for whom some costs would be covered by the sponsors, and therefore not captured in the databases. The extent to which any such information bias might the affect comparisons is unknown. Although patients in the MarketScan database are geographically diverse, they may not be representative of all patients in the US. In particular, individuals employed by small- to medium-sized employers are likely underrepresented in the commercial database, and patients enrolled in Medicare Advantage (Part C) plans (~31% of all Medicare beneficiaries) are largely absent from the Medicare database^20^. Although we attempted to control for baseline characteristics using inverse probability of treatment weighting, data on some clinical characteristics (e.g., bone marrow biopsy and serum protein test results) were unavailable and the possibility that comparisons between cohorts were confounded by unmeasured characteristics must be recognized. While the algorithm used to identify lines of therapy in this study was based on one used in prior studies^6,18^, the algorithm has not been clinically validated, and lines of therapy might have been misclassified in some instances. Progression was identified based on advancement to the next LOT, evidence of events indicative of progression, or evidence of death. While it was expected that these proxies captured the majority of progression events, their sensitivity and specificity are unknown.

## Conclusions

For MM patients receiving transplant and drug therapy, the economic burden of disease is greater among patients who progressed within 12 months of treatment initiation than those who do not, possibly highlighting the impact of early relapse and aggressive disease. This pattern was observed across all LOTs and time periods. Treatments that delay progression may yield important reductions in downstream disease management costs. These savings should be considered in frameworks assessing the value of innovative treatments for MM.

## Supplementary information

Appendix
